# Learning transitions–a descriptive study of nurses’ experiences during advanced level nursing education

**DOI:** 10.1186/s12912-015-0080-z

**Published:** 2015-05-12

**Authors:** Marit Graue, Bodil Rasmussen, Anne S. Iversen, Trisha Dunning

**Affiliations:** Faculty of Health and Social Sciences, Centre for Evidence-Based Practice, Bergen University College, 7030, , 5020 Bergen, Norway; Faculty of Health and Social Sciences, Institute of Nursing, Bergen University College, Bergen, Norway; Department of Paediatrics, Haukeland University Hospital, Bergen, Norway; School of Nursing and Midwifery, Faculty of Health, Deakin University, Melbourne, Australia; Centre for Nursing and Allied Health Research, Deakin University and Barwon Health, Geelong, Australia

**Keywords:** Learning transitions, Nursing education, Qualitative research, Diabetes nursing

## Abstract

**Background:**

Building capacity in a changing health care system is a challenge for advanced nursing education programs. Master-level nursing education is increasingly becoming the required education level for specialist nurses, and additional studies are needed to learn more about students’ experiences and learning transitions while undertaking such education. This study aimed to explore nursing students’ experience of their learning transitions while undertaking advanced nursing education and to describe how they translated the new knowledge and competence they gained into clinical practice.

**Methods:**

We used a qualitative research design with narrative self-reported reflections. 34 nurses (95 % women) from both urban and rural areas working with children, with adults in outpatient and inpatient endocrinology clinics in hospitals or with adults, including older people, attending primary health care services participated in the study. We collected data at two time points 15 months apart. Time one was the first week of the advanced nursing education, and time two was the completion of the education program. We used Malterud’s modification of Giorgi’s phenomenological analysis, otherwise known as systematic text condensation, to analyze the data.

**Results:**

Two core themes captured the participants’ experiences. The first theme was “assessing the situation of people with diabetes from a different perspective”, with the subthemes “an expanded perspective of practice and higher level of reflection”, “applying critical thinking in practice” and “changing patient-nurse relationships in diabetes care”. The second core theme was “a change in participants’ perception of their professional position”, with the subthemes “a greater knowledge base enhancing professional confidence” and “a more equal position within the professional team”.

**Conclusions:**

The study provides in-depth information about transition into advanced nursing education and can inform curriculum developers, nurse educators, policy-makers and nursing managers about how nursing education broadened participants’ perspectives of nursing and enhanced their confidence and professional position.

## Background

Building capacity and encouraging professional development that enable health professionals to work in a changing health care system are challenges for advanced nursing education programs. Caring for people with complex needs involves a team-based approach, and health professionals need the knowledge and attributes to communicate with health professionals from different disciplines and in different settings [1]. Recognition is growing that the biomedical paradigm of the last century is inadequate today and may hamper effective nurse-patient communication [2]. Specialist nurses need to be able to support people with chronic conditions, to plan care that encompasses the complexity of care and to raise the standard of care [3].

According to the International Diabetes Federation’s *IDF diabetes atlas* [4], 382 million people had diabetes in 2013. This number will increase to an estimated 592 million by 2035, which means that 1 in 10 adults will have diabetes. Nurses and other health professionals therefore need to be educationally prepared to obtain positions in which they can advocate for a better health system, to prevent diabetes and its associated complications [4]. Many people with diabetes have complex needs, because many have more than one coexisting condition, such as obesity, stroke and cardiovascular disease. In addition, adults with comorbidity have been reported to have a 1.3 to 4.6 times higher risk of having diabetes [5].

Master-level education is increasingly becoming the required education level for specialist nurses, but the direct benefit of advanced nursing education programs to care delivery or improved patient outcomes needs to be explored further [6]. The wider community and people with diabetes expect diabetes specialist nurses to be well informed and confident and to have a strong commitment to their profession as well as sound intellectual, interpersonal and clinical skills. In addition, clinical and professional activities and outcomes are needed to capture the leadership contribution in future evaluations of advanced nursing roles [7].

Attaining these attributes to improve diabetes care involves making the transition from a general nurse to an advanced or specialized diabetes nurse. Transitional experiences are complex and multidimensional [8, 9] and are crucial for self-growth and development [10]. Transitional experiences are foundational to how people learn [11]. How people in transition perceive, comprehend and respond to change [12] influences how they manage change and adapt to their new roles. In the current context, transition refers to changing from general nursing to advanced nursing. Moreover, people in transition need to be able to recognize turning points, which usually signify the beginning of a change in their life, and to adapt to the new situation.

Some studies report students’ experiences while undertaking master-level nursing education [13, 14]. The students in these studies highlighted the altered content and demands of the nursing education and indicated that it led to frustration and uncertainty but enabled the students to develop new ways of critical thinking and questioning. Additional studies are needed to further explore students’ learning transitions while undertaking nursing education programs. The current study therefore aimed:

- to explore nursing students’ experiences of their learning transitions while undertaking an advanced nursing education program; and
- to describe how they translated the new knowledge and competence they gained into clinical practice.

## Methods

We used a qualitative research design with narrative self-reported reflections [15]. The human experience in the current study focused on participants’ transition towards mastering advanced nursing skills as they moved from generalist to advanced practice [16] and as they adapted to and undertook a new professional role as advanced specialist nurses.

### Participants and sampling population

We used purposive sampling. All 35 students from both rural and urban areas of Norway taking the first 60 European Credit Transfer and Accumulation System (ECTS) credits of a 120 ECTS master-level nursing education program were provided with written information about the study. One student declined to participate. The nurses (*n* = 34, 95 % women) worked in a variety of settings including working with children, with adults in outpatient and inpatient endocrinology clinics in hospitals or with adults, including older people, attending primary health care services.

### Data collection

We collected data in the form of narrative self-reported reflections at two time points 15 months apart. Time one was the first week of the advanced nursing education (baseline), and time two was the completion of the education program. At baseline, the participants were invited to describe one actual patient situation they encountered in their clinical nursing role (Table 1). A group of course coordinators from various postgraduate courses over some years developed the guidelines for describing the situation. The reflection tool was refined regularly and was used among other student groups in training sessions on reflection communication. The tool effectively generated good discussions in groups. The tool was therefore considered appropriate to use to collect data in the current study. The participants included information about the clinical setting in which they worked and the nursing expertise required to provide diabetes care.

**Table 1:** Instruction guidelines for diabetes specialist nurses in writing reflection notes while undertaking an advanced nursing education program

Study start	End of study
Describe 1–2 specific clinical situations in which you had the opportunity to function as a diabetes specialist nurse. Think of a patient or a user with whom you have been in contact who has a problem or a need that requires your expertise as a diabetes nurse specialist. xYou may describe a situation that had successful outcomes or one in which the outcome was not so successful.	You have previously described a clinical situation in which you had the opportunity to function as a diabetes specialist nurse.
	You are now being asked to return to the same clinical case you have previously described and to reflect on this situation based on the knowledge you have obtained as a student in the first year of the nursing education program. It is not always easy to know how one would experience a given situation a second time, but we would like you to reflect as much as possible on the issues below and to describe what you consider would be most realistic for you now if you were to experience this situation again.
Part 1	Part 1
Briefly describe the patient or user and his or her life situation (make it anonymous).	The same case described previously.
Describe the patient’s problems or needs.	
What happened or is happening with the patient or user in relation to the problem or need?	
When did the problem or need arise?	
Who was involved in the situation when the problem or need arose?	
In what way or ways were or are you involved in the problem or need?	
What action was carried out in relation to the problem or need?	
How do you evaluate the help that the patient or user was given?	
What were or are the most obvious nursing and health challenges concerning the problem or need?	
What would you do differently if you faced the same situation again?	
Part 2	Part 2
What are the specific factors in the situation you have described that make it important for you as a diabetes specialist nurse to be involved?	What would be your thoughts concerning this same situation now that you have undertaken the diabetes nursing education program?
What was it specifically about the situation(s) or the patient or user you have described that made it a source of learning for you?	How would you feel in this same situation now that you have undertaken the diabetes nursing education program?
What did you learn from the experience you have described?	How would you act in this same situation now that you have undertaken the diabetes nursing education program?
	What would be similar and why?
	What would be different and why?
Part 3	Part 3
In which areas do you feel you need to develop your own professional and personal expertise?	In what ways has being a student in diabetes nursing education influenced your competence as a professional and also you as a person?
What specific goals do you have for acquiring the learning you need to further develop your expertise as a diabetes specialist nurse?	
What do you feel you need to achieve these goals?	

At the end of the nursing education program, we asked participants to reflect on the patient they wrote about at baseline. We encouraged participants to reflect on and describe how their newly acquired knowledge would influence the diabetes care they described at baseline and how new knowledge would help them to resolve the diabetes care challenges they encountered at that time (Table 1). We grouped the data from each student sequentially to capture the essence of their reflections on their learning trajectory. All quotes used in the findings are from the second time point and reflected changes in participants’ self-reported learning and clinical decision-making from the first time point.

Students wrote their reflections in Norwegian. Most used their own computers at home. Only a few students wrote their reflections by hand. Each student wrote two to three pages. The students submitted their reflections to the researchers in a sealed envelope without identification. The researchers delivered them to a research assistant, who translated them from Norwegian into English. The research assistant is Norwegian and speaks and reads English fluently.

After translation, we organized the English and Norwegian versions in parallel columns in the same document and performed data analysis using both texts. One of the Australian researchers (BR) ensured the accuracy and consistency of the translated text. BR is a Dane, has family in Denmark and in Norway and speaks and understands both languages fluently. She has lived and worked in Australia for 30 years and speaks and reads English fluently. MG and ASI are native Norwegians and read and write English daily in their professional roles as researchers.

### Data analysis

We used Malterud’s [17] modification of Giorgi’s phenomenological analysis, otherwise known as systematic text condensation, to analyze the data. The method comprises four stages: 1) gaining an overall impression of the data by reading the entire description and searching for emerging themes; 2) identifying and sorting units of meaning: organizing the parts of the text to be explored more closely by identifying and coding units of meaning that relate to the themes that emerged in step one; 3) condensing and organizing the coded units of meaning into groups according to their code, sorting them into subgroups and condensing the content of the subgroups in the form of abstract meaning; and 4) synthesizing by summarizing or describing subgroups as analytical text, noting concepts in the information abstracted from each coded unit of meaning and their subgroups, including citations from the written texts [17].

We confirmed and substantiated the findings by examining the coded units of meaning and the emerging themes with each individual participant’s data and then comparing them with other participants’ data. MG and BR undertook the cross-comparison and searched the data systematically for other important data that did not fit the emerging core themes and subthemes. All authors then compared the themes and synthesized them into meaningful core and subthemes.

### Validity and rigor

Qualitative research needs to be rigorous and must therefore be transparent and explicit. In this study, we enhanced validity by involving independent external experts with advanced knowledge and experience in diabetes education: TD (credentialed diabetes educator and recognized international and national diabetes researcher and curriculum developer) and BR (course leader and nationally recognized diabetes curriculum developer). ASI (course leader and curriculum developer for advanced pediatric nursing education) and MG (course leader and curriculum developer for advanced diabetes nursing education) are from Norway. Three researchers (BR, ASI and MG) independently read the nurses’ reflections in Norwegian, and all four researchers read the English translation, analyzed the data independently and then discussed their interpretations to reach consensus.

### Ethical issues

Ethics approval was granted according to Norwegian law and processes, which only required informed consent for anonymous data about nurses’ experiences and their learning trajectories. We gave the participants oral and written information about the project before they provided consent. We informed participants that all information would be treated confidentially and that their right to privacy would be protected by deidentifying data and using codes when reporting the findings. They were informed that they could withdraw from the study at any time during the ongoing nursing education without any consequences to them. Data from the two time points were deidentified at the end of the nursing education program, and personal identification was no longer possible.

MG was involved as the course co-coordinator and lecturer, and the students therefore knew her. However, the reflection notes were anonymous, and only the transcriber (the research assistant) could identify participants to connect the reflection notes from the two time points. None of the other three researchers were directly involved with the participants.

## Results

The learning transitions identified in the study were complex and individual. However, two core themes clearly captured the learning transitions participants experienced while undertaking the advanced nursing education program. The first core theme was “assessing the situation of people with diabetes from a different perspective”. The second core theme was “a change in participants’ perception of their professional position”.

### Assessing the situation of people with diabetes from a different perspective

#### An expanded perspective of practice and higher level of reflection

The participants indicated that they were more aware of the importance of new knowledge in their work and more likely to consider the evidence when providing care after finishing the advanced nursing education program. For example, they felt that the newly acquired knowledge gave them an expanded perspective of practice that enhanced their ability to solve problems and make decisions, enhanced their communication with people with diabetes and resulted in safer care. One participant’s reflection highlights how the education enhanced communication skills.

Having to reflect on how I communicate and act in a consultation [with patients] … has given me some eye-opening moments, and I wish to work more with this and further develop [my skills]. [N3]

Enhanced ability to communicate was evident in participants’ comments about how they reflected on their actions more frequently than before undertaking the advanced nursing education program. They reported that their enhanced communication skills enabled them to provide more patient-centered care and to work in partnership with people with diabetes.

I think I feel empowered in these situations [communication with people with diabetes] today. It is not me who is supposed to fix the patients’ problems. I see myself more as a collaborative partner or companion. Today I would more thoroughly have explored her coping resources and mapped her motivation. Let her control the aims to a larger extent. [N6]

Participants clearly articulated the learning transition by reporting that the nursing education program better equipped them to use active and reflective listening skills to build rapport with people with diabetes.

#### Applying critical thinking in practice

Another major learning transition was the participants’ ability to use evidence-based literature and to think critically. These newly acquired skills and knowledge helped participants to navigate databases, use evidence-based guidelines and distinguish evidence-based from non-evidence-based information. The latter is essential to their ability to evaluate and set priorities among care strategies.

Accomplishing this nursing education program has made me more confident both as a person and as a professional … Being taught methods and evidence-based practice has helped me find and use research-based knowledge. [N1]

One participant said that thinking critically enabled her to provide safer practice and was very satisfying.

Applying evidence-based knowledge in practice gives more professional strength and safety for the patients and also provides meaning and satisfaction for me personally. [N17]

The participants described how they transferred the knowledge they obtained in the nursing education program into clinical practice. They also explained how their ability to solve problems enhanced their clinical thinking. One participant described critical thinking as a tool that helps to make research databases more relevant to practice.

Professionally I have grown a lot. I have been given a lot of new tools I can use to keep myself professionally updated. Research databases and the ability to evaluate the information there are worth their weight in gold. [N8]

Likewise, participants described how critical thinking and being able to identify relevant evidence improved their understanding of research. One participant explained how she applied her new research skills in practice.

The DAWN study found that 41 % of the patients in the study had poor mental health and only 10 % reported that they were being treated for it. I would now [after the nursing education program] be more conscious about mapping [a patient’s] entire situation, and clarify thoughts and feelings, and together with [the patient] try to find a specific plan for further care and treatment. [N17]

#### Changing patient–nurse relationships in diabetes care

Participants explained that the nursing education program changed how they established relationships with people with diabetes. For example, participants said their improved ability to use evidence enhances their relationships with patients by shifting the power in the relationship so that the people with diabetes feel empowered to express their needs. Empowerment emerged as a central concept in participant’s descriptions of the patient–nurse relationship. Empowerment represented a learning transition, since the participant’s perception of their professional roles encompassed a change to a higher level of autonomy and clarity related to the strategies they used in practice. Enhanced patient empowerment was an outcome of their learning but also resulted in a sense of growth in their own empowerment. One participant described how they applied empowerment to practice by listening more and giving less advice.

… I do not think I lacked understanding of the total situation of the patient [when starting the nursing education program] … but I had a clear opinion on what was important for the patient to give priority to, and in this way I set the goal for the patient. If I were to do something differently [now] …, it would be listening more and giving less advice. The central issue in empowerment thinking is that patients need to set their own goals. Within this perspective, our role as health professionals is to listen actively and to help the patients clarify problems regarding their own care, feelings and goals. [N3]

A significant learning transition was a change in participants’ perspective from patient care requiring them to be “expert problem-solvers” to understanding the importance of developing a collaborative partnership approach. The change represented a major shift in participants’ thinking.

Most participants felt that the change in the patient–nurse relationship occurred because they changed their decision-making processes when they learned about the needs and coping strategies of people with diabetes.

Previously I was more concerned about having a solution or answer to give the patient, and because of this I often confronted the patients in conversations. Now I see that the patient often has the solution, and communication techniques where the patient’s own resources or knowledge is revealed provide better conversations and solutions. [N28]

### A change in participants’ perception of their professional position

#### A greater knowledge base enhancing professional confidence

Participants indicated a major learning transition was moving from uncertainty to greater certainty in their personal ability to develop effective care strategies. The transition was a painful process because it was accompanied by self-doubt. However, as participants’ confidence increased, they began to take pride in their personal and professional development. Participants coped with the uncertainty by realizing they were becoming empowered and sought opportunities to demonstrate their knowledge of diabetes and professional confidence.

By delving into such essential terms as health, health-promoting work, salutogenesis and empowerment, I have been able to anchor the knowledge acquired through practice on a more solid platform that is good to have further on. I experience that the meaning of these terms resonates with the way I wish to work. [N3]

Participants indicated that their ability to apply reasoning and problem-solving to clinical care enhanced their professional confidence and clinical reasoning.

Confidence is the first word that strikes me when thinking about what this further education has given me. The knowledge I have gained definitely makes me more self-confident as a diabetes specialist nurse. It is more fun to have diabetes consultations! I dare to express my opinion in various situations because I know so much more. One is much more assured in communicating with the patient, and one gets the feeling that what one says has more professional weight. I think a lot about how important it is to provide evidence-based knowledge. [N4]

The participants reported that applying evidence to practice enhanced their professional power. This consequently improved their level of professional satisfaction. They gained a clearer perspective of the focus of diabetes nursing after undertaking the nursing education program and realized how advanced nursing differs from general nursing care.

In general I have increased my knowledge, competence and confidence related to taking care of people with diabetes. It has also made it easier for me to make myself and my knowledge more visible, both in relation to other occupational groups and to people with diabetes. [N1]

#### A more equal position within the professional team

Several participants explained that their newfound confidence and competence enhanced their interactions with other health professionals. This led to increased recognition of their professional position and respect by other health professionals. The participants also indicated the advanced nursing education program enhanced self-awareness of their role in the diabetes team.

The studies in the nursing education program have affected me positively both personally and professionally. I feel stronger and I experience that other people see me as a resource person on the ward at which I work. [N8]

I have achieved my goals on being able to work evidence-based . … I already see that this has resulted in me being included in the development work in my workplace and that the relationship with the specialists with whom I work has become more equal. [N13]

Another participant said she felt more empowered to make a difference at an organizational level.

Through several years of struggling as a nurse in the bureaucratic system under which we work, I have often felt that there is no use trying to change things or I have just given up and followed earlier recommendations. This way of thinking has gradually changed during the nursing education program. The things you are excited about as a nurse, also at the organizational level, are possible to increase focus on, start discussing and checking databases for research and guidelines and perhaps change via a common effort. [N7]

## Discussion

The findings suggested that an advanced nursing education program based on the principles of evidence-based practice can bring about learning transitions. This enabled diabetes specialist nurses to respond more effectively to the many challenges in contemporary diabetes health care. The current study identified and described how personal and professional competence can grow when a nursing education program meets the students’ learning needs and encompasses reflective practice. Learning transitions bring a new perspective to understanding the academic and personal factors that influence the transition from general to advanced clinical practice.

The participants gave examples to illustrate how they began to appreciate each person’s individual situation in a more holistic way by using the effective communication strategies they acquired during the nursing education program. Recent results from the DAWN2 study [18] showed that assessing psychosocial needs is essential to improving outcomes for people with diabetes. Making a successful learning transition to focus more on the psychosocial aspects of care depends on the individual’s ability to reflect on relevant issues and emotions and awareness of and insight into the significance of the change [11]. The current participants’ reflections after the advanced nursing education program clearly show how reflection on learning enabled them to better negotiate the transition from general nurses to diabetes specialist nurses.

The global workforce needs to be educationally prepared to meet the complex social and mental challenges involved in helping people with diabetes to self-manage their condition [1]. Our findings highlight that some of the learning transitions involve student engagement at the practical, academic and, importantly, emotional levels. Preparing nurses to meet challenges on all three levels will help them in making the transition into advanced practice roles, particularly when the student is prepared to meet these challenges early in the nursing education program. The current participants described some positive outcomes of their transition to advanced practice. These included deeper understanding of their professional skills and a change in their perception of their professional position within the health care team.

Diabetes care is a longitudinal process, and a collaborative infrastructure and integrated care teams are key to meeting the needs of patients. At the system level, larger interdisciplinary care teams are being advocated [19]. Participants perceived themselves as being more visible in the interdisciplinary team at the end of the nursing education program. They were not only aware but also able to describe the transition of their professional position moving to a different level during the advanced nursing education program. Awareness of this shift in role is a part of transitions, since they comprise several essential properties [10, 20]. Awareness is a defining characteristic of transition, and the person must be aware of the changes that are occurring [8]. It is essential that people let go of the old and known to make a successful transition and to move on [11]. According to Maten-Speksnijder et al. [21] offering learning opportunities to reflect on experiences related to the nurse–patient interface and about role development and role transitions in master programs is essential.

The participants described how the advanced nursing education program changed their communication skills, care planning methods and perception of professional roles: that is, their transition to advanced practice roles. Transitions in the current study describe a process of change ranging from initial uncertainty to becoming more confident that, in turn, changed the participants’ professional position both within the team and in the organization. The sense of changed professional position is clearly an outcome of a transition elicited by the advanced nursing education program. With the changed perception of themselves as professionals, the participants also experienced that patients and health professionals in their professional networks responded differently to them, which resulted in a sense of new professional identity.

Wackerhausen [22] described two levels of professional identity: a macro and a micro level. The macro level is the public face of a profession, combining official recognition such as recognition of qualifications, regulations and duties, the public perception of the profession, the related professions’ views (for example nursing and medicine) as well as the self-image the profession’s leaders promote. The micro level is described as a set of qualities a person must have to be a fully recognized member of the profession: for example he or she must behave according to the cultural dimension of the profession. This is not set by any formal education but more like unarticulated etiquette, metaphorically described as being “one of our kind” [22]. These two levels are identifiable in our data, particularly the micro-level identity related to interprofessional relationships. Participants perceived that their newly acquired ability to act as patient advocates and apply evidence to professional practice was a turning point in their professional development that led to professional recognition from other health professionals and enhanced their pride in their ability and confidence in their specialist role. They felt they could make a difference. These factors are clear signs of a positive transition to advanced nursing.

Participants in the current study gave many examples of how the nursing education program enhanced their ability to practice at an advanced level, including identifying and evaluating research and applying it in clinical practice. They indicated that critical appraisal skills helped them to be more aware of the importance of using evidence in clinical decision-making. Well-trained health professionals equipped with high-quality evidence are required to provide high-quality evidence-based health care and keep patients safe [23]. The current participants explained that they became more aware that their reasoning should be based on the best evidence and how to use evidence in their work. Clearly, the nursing education program supported participants through the transition into advanced practice and helped them to acquire the necessary concepts and theories to engage in communication with other health professionals.

### Limitations

This study had several limitations. First, using nurses’ self-reported reflections around one clinical situation at two time points 15 months apart may not have identified other aspects of advanced practice nursing, and further perceptions of their personal and professional development may have been set aside. Second, the study was limited to nurses undertaking advanced nursing education in Norway. This could be seen as a weakness, since the curriculum and context may differ from advanced nursing education programs in other countries. Third, the study did not represent the perceptions of patients or other team members. Such data could have enhanced our understanding of patient–nurse communication and provided additional insights into team interactions and nurses’ ability to take on leadership roles. A further limitation was that participants reported mainly positive learning transitions. One explanation for this was that the participants were asked to reflect around the same clinical situation they had previously described based on the knowledge they had obtained while undertaking an advanced nursing education program. Any negative perceptions and experiences of participants may have been less stimulated, since the intent of the study was to reveal how they translated the new knowledge and competence they obtained into clinical practice.

## Conclusions

The current study demonstrates how evidence-based knowledge and use of reflection tools can improve patient–nurse relationships, inform clinical decision-making and facilitate increased confidence and pride in the profession within interprofessional diabetes teams. We recommend using patient cases in advanced nursing education programs to link theory and practice with research evidence to achieve optimal patient-centered care. Reflection tools should be integrated into future advanced nursing education programs. The study provided in-depth information about transition to advanced nursing and informs curriculum developers, nurse educators, policy-makers and nursing managers how nursing education programs can broaden participants’ perspectives of diabetes nursing and enhance their confidence and professional position. Advanced nursing education programs are critical to developing professional identity and competence.
